# FUNCTIONAL CAPACITY IN CHILDREN AND ADOLESCENTS WITH CONGENITAL HEART DISEASE

**DOI:** 10.1590/1984-0462/;2019;37;1;00016

**Published:** 2019-01-07

**Authors:** Camila Wohlgemuth Schaan, Gabriela Feltez, Beatriz D’Agord Schaan, Lucia Campos Pellanda

**Affiliations:** a Instituto de Cardiologia do Rio Grande do Sul/Fundação Universitária de Cardiologia, Porto Alegre, RS, Brasil.; b Universidade Federal do Rio Grande do Sul, Porto Alegre, RS, Brasil.; c Universidade Federal de Ciências da Saúde de Porto Alegre, RS, Porto Alegre, Brasil.

**Keywords:** Heart defects, congenital, Exercise tolerance, Child, Adolescent, Cardiopatias congênitas, Tolerância ao exercício, Criança, Adolescente

## Abstract

**Objective:**

To evaluate the physical activity level and functional capacity of children and adolescents with congenital heart disease and to describe correlations between functionality, surgical and echocardiographic findings, metabolic and inflammatory profile and differences between acyanotic and cyanotic heart defects.

**Methods:**

A cross-sectional study including children and adolescents with congenital heart disease between six and 18 years old that were evaluated with the 6-minute walk test (6MWT) to assess functional capacity. The short version form of the International Physical Activity Questionnaire (IPAQ) was performed to evaluate physical activity levels. Also, echocardiography and blood collection, to evaluate the metabolic (blood glucose, lipids, insulin) and inflammatory markers (C-reactive protein), were assessed.

**Results:**

Twenty-five individuals were evaluated. Of them, 14 had acyanotic heart defects and 11 cyanotic heart defects. Mean age was 12.0±3.7 years, and 20 (80%) were male. IPAQ showed that six (24%) individuals were very active, eight (32%) were active, nine (36%) had irregular physical activity, and two (8%) were sedentary. The mean distance walked in the 6MWT, considering all studied individuals, was 464.7±100.4 m, which was 181.4±42.0 m less than the predicted (p=0.005). There was a positive correlation between Z score 6MWT and the number of surgical procedures (r=-0.455; p=0.022).

**Conclusions:**

Children and adolescents with congenital heart disease have low functional capacity, but they are not completely sedentary.

## RESUMO

**Objetivo:**

Avaliar o nível de atividade física e a capacidade funcional de crianças e adolescentes com cardiopatia congênita, além de descrever correlações entre funcionalidade, achados cirúrgicos e ecocardiográficos, perfil metabólico e inflamatório e diferenças entre cardiopatias congênitas acianótica e cianótica.

**Métodos:**

Estudo transversal com crianças e adolescentes com cardiopatia congênita entre seis e 18 anos de idade. Foi realizado o teste de caminhada de 6 minutos para avaliar a capacidade funcional, e aplicou-se a versão curta do Questionário Internacional de Atividade Física (IPAQ) para avaliar os níveis de atividade física. Foram feitos também: exame ecocardiográfico, coleta de sangue para avaliação de perfil metabólico e inflamatório (glicemia, triglicerídeos, colesterol total, lipoproteína de alta densidade - HDL-colesterol, lipoproteína de baixa densidade - LDL-colesterol, hemograma completo, proteína C reativa, insulina).

**Resultados:**

Foram avaliados 25 indivíduos, dos quais 14 tinham cardiopatia congênita acianótica e 11 cianótica. A média de idade foi de 12,0±3,7 anos, e 20 (80%) eram do sexo masculino. O IPAQ mostrou que seis (24%) indivíduos eram muito ativos, oito (32%) eram ativos, nove (36%) tinham atividade física irregular e dois (8%) eram sedentários. A média de distância percorrida no teste de caminhada dos 6 minutos, considerando todos os indivíduos estudados, foi de 464,7±100,4 m, sendo 181,4±42,0 m menor do que o previsto (p=0,005). Encontrou-se correlação entre o escore Z do teste de caminhada de 6 minutos e o número de procedimentos cirúrgicos realizados (r=-0,455; p=0,022).

**Conclusões:**

Crianças e adolescentes com cardiopatia congênita têm baixa capacidade funcional, mas não são completamente sedentários.

## INTRODUCTION

With advances in surgical techniques and postoperative support, a significant increase in survival of children with congenital heart disease (CHD) has been observed. However, there was a consequent increase in long-term morbidity, due to surgical sequelae, inflammatory responses associated with cardiopulmonary bypass,^1^ and development of inadequate health habits later in childhood, added to the initial under nutrition and exercise restrictions.^2^ ^,^ ^3^

Physical inactivity is associated with increased risk of coronary heart disease, and healthy physical activity habits are established during childhood. It has been shown that regular, organized and early physical activity in schools leads to tracking of healthy physical activity habits into adulthood.^4^ Children and adolescents with CHD tend to be sedentary, with low levels of physical activity when compared to healthy individuals,^5^ ^,^ ^6^ and more often become sedentary adults, with risk factors for coronary heart disease.^7^

Inflammatory markers and metabolic derangements are changes expected, especially in children and adolescents with excessive weight.^8^ Physicians themselves may recommend these children not to engage in physical activities, which results in a higher risk of obesity.^9^ However, even when there is no medical recommendation to limit physical activities, parents themselves take this initiative, for fear of events such as arrhythmias or sudden death.^10^

Low levels of physical activity are directly related to functional capacity.^11^ ^,^ ^12^ It is important to assess physical activity level and functional capacity in CHD children and adolescents, to allow the application of preventive health measures early during the life course.^6^ ^,^ ^7^ ^,^ ^13^ However, few studies use the 6-minute walk test (6MWT) for functional capacity evaluation in the pediatric population with CHD. The 6MWT is a submaximal, low-cost test that is easy to apply and is mainly indicated for measuring response to treatments, but also used as a predictor of morbidity in other populations.^14^ ^,^ ^15^

Thus, the objective of the present study was to evaluate the physical activity level and the functional capacity of children and adolescents with CHD using the 6MWT. We also investigated the existence of possible correlations of functional capacity with surgical and echocardiographic findings, metabolic and inflammatory profile and differences between patients with acyanotic and cyanotic CHD.

## METHOD

This was a cross-sectional study with children and adolescents between 6 and 18 years old, with medical diagnosis of CHD, followed at the Pediatric Cardiology Outpatient Clinic of a referral hospital in south of Brazil between February 2011 and March 2012. The study was approved by the institution’s Research Ethics Committee, and patients or guardians signed a written informed consent form. Exclusion criteria were: lower limb malformations, wheelchair use, and neurological sequels or other medical conditions that would prevent the proposed evaluations.

Initially, an evaluation form including data about underlying disease, previous surgeries or the presence of residual lesion was completed. Data were collected using a questionnaire administered by health professionals who attended specific training. Weight was measured to the nearest 0.1 kg, and height to the nearest centimeter, using an electronic digital scale with stadiometer (Welmy, São Paulo, Brazil) with 200-kg capacity, with the child standing, without shoes or heavy clothing. Nutritional status was based on body mass index (BMI), and classified using the software WHO Anthro and AnthroPlus (Geneva, Switzerland).

The short version of the International Physical Activity Questionnaire (IPAQ), validated in Brazil, which assesses physical activity level, was applied by a health professional specifically trained for this task. The questions are related to the time spent walking as a means of locomotion, performing vigorous and moderate activity. As a result, patients are classified as very active, active, irregularly active or sedentary.^16^

The 6MWT was conducted on a 20-meter corridor marked at each meter. Participants were accompanied by the researchers and stimulated by standardized verbal commands to walk over the longest possible distance within 6 minutes, according to the American Thoracic Society (ATS) guidelines.^17^ They were instructed to discontinue the test in case of any discomfort. At the beginning and at the end of the test, and after one minute of rest, the respiratory rate was measured, and dyspnea and fatigue of the lower limbs were evaluated through a colored modified Borg scale,^18^ graduated from 0 to 10 (in which 0 was no effort, represented in blue; and 10, the maximum effort that the participant could exert, represented in red). Blood pressure was determined at the beginning and at the end of the test with aneroid sphygmomanometers graduated from 0 to 300 mmHg. During the test, heart rate and peripheral oxygen saturation were measured every minute with a pulse oximeter (Onyx 9550 Nonin, Minnesota, United States). After completion of the test, the total distance walked by the patient was determined in meters. The walked distance during the test was compared with reference values and expressed as percentage of the predicted distance. Also, the values were transformed into Z scores, using the following formula: value found-normal value/standard deviation.^19^

Blood samples were collected after a 12-hour fast. Laboratory examinations included blood glucose (enzymatic method, Roche, Mannheim, Germany), triglycerides (enzymatic method, Roche), total cholesterol (enzymatic method, Roche), high-density lipoprotein (HDL) cholesterol (enzymatic method, Roche), low-density lipoprotein (LDL) cholesterol (enzymatic method, Roche), complete blood count (flow cytometry, impedance and direct fluorescence, São José dos Pinhais, Brazil), C-reactive protein (turbidimetry, Roche), and insulin levels (electrochemiluminescence, Roche).

All patients underwent a comprehensive transthoracic echocardiographic study, at rest in the supine position, with conventional methods. The end diastolic and systolic diameters were measured through the internal dimensions of the left ventricular cavity, obtained by two-dimensional and M-mode images from the parasternal window, with longitudinal viewing. The left ventricular ejection fraction was estimated by the Teichholz’s formula,^20^ according to the recommendations of the European Society of Cardiovascular Imaging (EACVI) and the American Society of Echocardiography.^21^ Left ventricular mass was calculated using the Devereux equation and then indexed by body surface area. There were two losses in the cyanotic CHD group, due to inadequate transthoracic window and consequent incomplete echocardiographic records of the measures used for the present study.^21^

The categorical variables were described as frequencies, and numerical variables as mean and standard deviation (normal distribution) or as median and 25-75 percentiles (non-normal distribution). A sample size of 25 was estimated based in the difference of 180 m between predicted and walked distance observed by Moalla et al.^22^ Considering the significance level of 0.5 and 80% of power, it would be necessary to study eight individuals in each group. We added a margin of 40% to allow for the great variability between individual heart lesions and patients age. The sample was divided into cyanotic and acyanotic CHD groups, for comparison of metabolic and inflammatory profile, echocardiography and 6MWT variables.

Independent sample Student’s t-test and Mann-Whitney U test were used to compare means between groups, according to the data distribution. The associations between physical activity levels determined by the IPAQ, distance walked in the 6MWT, echocardiographic findings and metabolic and inflammatory profiles were evaluated with Spearman’s or Pearson correlation coefficients. McNemar’s test was used to compare the perception of dyspnea and fatigue by the Borg scale between the groups (cyanotic versus acyanotic CHD), in which we compared the prevalence of zeros for fatigue and dyspnea at the beginning and at the end of the 6MWT. The analyses were performed using the software Statistical Package for the Social Sciences (SPSS) version 14.0 (IBM, Chicago, United States), and the significance level in all analyses was 5%.

## RESULTS

The sample was composed of 25 individuals, predominantly male (80%), mean age 12.0±3.7 years. Fourteen individuals had acyanotic CHD and 11 cyanotic CHD. Clinical data, anthropometric variables and levels of physical activity assessed by IPAQ are exposed in Table 1.

**Table 1:** Characteristics of patients with congenital heart disease.

	Total group (n=25)
Age (years)	12.0±3.7
Male	20 (80)
Weight (kg)	43.4±18.0
Height (m)	1.51±0.20
BMI percentile	48.7±26.6
Birth weight (kg)	3.127±0.6
Birth length (cm)	49.7±6.0
ECC total time (min)	63.0±43.7
Age at definitive surgery (months)	42.0±46.1
SBP (mmHg)	110.0±13.6
DBP (mmHg)	70.0±9.8
Schooling	
Incomplete basic level n (%)	23 (92)
Incomplete medium level n (%)	2 (8)
Gestational age	
Preterm n (%)	7 (26.9)
Number of surgeries	
None n (%)	4 (16)
1 n (%)	14 (56)
2 n (%)	5 (20)
3 n (%)	2 (8)
Residual injury n (%)	10 (40)
IPAQ n (%)	
Very active n (%)	6 (24)
Active n (%)	8 (32)
Irregular activity n (%)	9 (36)
Sedentary n (%)	2 (8)

Data expressed as mean and standard deviation (SD) or n and percentage in parenthesis; BMI: body mass index; ECC: extracorporeal circulation; SBP: systolic blood pressure; DBP: diastolic blood pressure; IPAQ: International Physical Activity Questionnaire.

The most frequent acyanotic CHD was interventricular and interatrial communication, in three individuals, followed by the atrioventricular septal defect and pulmonary stenosis, in two patients each. Other heart defects in this group, included coarctation of the aorta, bicuspid aortic valve, aneurysm of the sinus of Valsalva and aortic stenosis/regurgitation, affecting one individual each. In the cyanotic CHD group, the most common lesion was tetralogy of Fallot (ToF), affecting seven individuals, followed by truncus arteriosus, double outlet right ventricle, transposition of the great arteries and double inlet left ventricle, affecting one patient each. All seven patients with ToF underwent total surgical correction; one needed a Blalock-Thomas-Taussig shunt before total correction. The patient with transposition of the great arteries underwent arterial switch (Jatene procedure). One of the patients with pulmonary stenosis needed an aortic homograft. The two patients with univentricular heart underwent Glenn procedure followed by Fontan operation.

**Figure 1:**
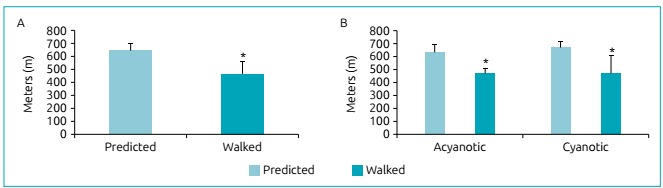
(A) Distance walked in the 6-minute walk test in all patients. Data expressed as mean and standard deviation. *p=0.005 (Student’s t-test); (B) distance walked in the 6MWT according to acyanotic or cyanotic group. Data expressed as mean and standard deviation. *p<0.001 versus predicted for acyanotic and cyanotic groups (Student’s t-test).

The functional capacity of the patients, evaluated by the distance walked in the 6MWT, is presented in Figure 1. Panel A shows that the mean distance walked by all patients (n=25) was 464.7±100.4 m, corresponding to 71.9±14.4% of the value predicted considering age, sex and height (p=0.05). Panel B presents the distance walked by patients from the cyanotic and acyanotic CHD groups. The results show that the functional capacity was lower than the one predicted in both groups (p<0.001).

Other results from the 6MWT are presented in Table 2. Two patients from the cyanotic CHD group discontinued the test due to lower limb fatigue. An increase of heart rate and respiratory rate was observed in both groups after the 6MWT, and increased systolic blood pressure was seen only in the acyanotic CHD group.

**Table 2:** Variables related to functional capacity and physical activity level according to the presence of cyanotic or acyanotic congenital heart disease.

	Acyanotic CHD (n=14)			Cyanotic CHD (n=11)		p-value
6MWT						
Distance walked (m)	464.7±49.6			464.7±144.9		1.000
Z score	-2.5±1.0			-3.1±1.9		0.395
	Initial	Final	p-value	Initial	Final	p-value
HR (bpm)	80.3±14.9	107.0±25.2	<0.001	77.8±22.4	103.0±16.6	0.001
RR (mrpm)	19.6±2.4	24.4±3.8	<0.001	17.2±4	21.7±3.5	<0.001
SpO_2_ (%)	98.6±1.3	97.8±1.3	0.216	95.4±4.5	92.4±5.8	0.120
SBP (mmHg)	102.3±14.3	111.4±10.9	0.034	112.9±17.9	118.2±17.7	0.300
DBP (mmHg)	63.5±8.4	68.2±8.3	0.121	77.4±11.1	73.6±12.8	0.380
Borg dyspnea	11 (78.5)	9 (64.2)	0.500	7 (63.6)	5 (45.5)	0.625
Borg fatigue	13 (92.8)	13 (92.8)	1	11 (100)	6 (54.5)	0.708

CHD: congenital heart disease; 6MWT: 6-minute walk test; HR: heart rate; RR: respiratory rate; SpO_2_: peripheral oxygen saturation; SBP: systolic blood pressure; DBP: diastolic blood pressure; data expressed as mean and standard deviation (SD), n and percentage in parenthesis or median and percentage in parenthesis; McNemar Test.

Table 3 presents the metabolic and lipid profile of the patients, which were within the normal range for the age group. HDL cholesterol levels were lower (p=0.003), and C-reactive protein levels were higher in the cyanotic CHD group (p=0.009). Echocardiographic results are also presented in Table 3. The left ventricular diastolic diameter and the right ventricle diameter were smaller in the acyanotic CHD group.

**Table 3:** Laboratory and echocardiographic findings in the group and according to the presence of cyanotic or acyanotic congenital heart disease.

	Total group (n=25)	Acyanotic CHD (n=14)	Cyanotic CHD (n=11)	p-value*
Laboratory				
Total cholesterol (mg/dL)	144.8±24.2	150.7±22.0	137.1±25.8	0.169
HDL-cholesterol (mg/dL)	51.2±13.7	58.0±14.2	42.5±6.6	0.003
LDL-cholesterol (mg/dL)	76.9±21.4	77.3±22.9	76.4±20.4	0.923
Triglycerides (mg/dL)	66.0 (50.0-118.0)	63.5 (49.2-116.7)	67.0 (49-122)	0.893
Glycemia (mg/dL)	89.1±8.9	88.0±6.6	90.0±11.4	0.521
Insulinemia (mg/dL)	6.3 (4.3-8.3)	5.5 (2.8-8.0)	6.6 (4.7-12.4)	0.222
CRP (mg/dL)	0.06 (0.03-0.17)	0.04 (0.02-0.06)	0.14 (0.06-0.28)	0.009
Hematocrit (%)	39.4±3.5	38.2±3.6	40.9±3.0	0.069
Hemoglobin (g/dL)	13.3±1.35	12.9±1.3	13.9±1.1	0.071
Echocardiographic				
Ejection fraction (%)	66.0±16.9	64.8±18.4	67.9±15.0	0.676
Delta D (%)	38.7±16.9	38.0 (30.3-44.9)	38.0 (30.3-41.9)	0.925
LV systolic diameter (cm)	2.6 (1.9-3.1)	2.5 (1.9-2.9)	2.9 (2.4-3.1)	0.216
LV diastolic diameter (cm)	4.2 (3.4-4.7)	3.8 (3.3-4.3)	4.7 (4.2-4.9)	0.005
RV diameter (cm)	2.1 (1.5-3.0)	1.7 (1.3-2.1)	3.0 (2.2-4.9)	0.016

CHD: congenital heart disease; HLD: high-density lipoprotein; LDL: low-density lipoprotein; CRP: C-reactive protein; LV: left ventricle; RV: right ventricle; data expressed as mean and standard deviation (Student’s t-test); median and 25-75 percentile (Mann-Whitney U test); *comparison between acyanotic and cyanotic groups.

No correlation was found between the distance walked in the 6MWT and extracorporeal circulation total time ­(r=-0.012; p=0.955), age of definitive surgery (r=0.154; p=0.506), ejection fraction (r=0.304; p=0.154) and C-reactive protein (r=-0.225; p=279). The correlation of the 6MWT and echocardiographic, surgical and laboratory data showed that patients who underwent less surgical procedures presented Z scores for 6MWT (r=-0.455; p=0.022).

## DISCUSSION

This study with CHD patients showed that a low percentage of children and adolescents is sedentary, according to the IPAQ, but their functional capacity is reduced, compared to that predicted. The functional capacity was similar in patients with cyanotic or acyanotic CHD.

The mean distance walked in the 6MWT in the present study was 181.4 m shorter than predicted by gender, height, and age, which is in agreement with other studies,^23^ although another report^22^ showed a smaller difference between observed and predicted. The differences could be due to the small number of individuals evaluated in the study of Feltez et al.,^23^ as well as age, since only adolescents between 12 to 16 years old were included in that study. Furthermore, no test interruption was reported in that study, differently from the present one, increasing the difference between predicted and observed.

Although only 8% of children and adolescents included in this study were sedentary, the frequency of very active individuals was low (24%). In an investigation of the level of physical activity in 153 adolescents with CHD, Lunt et al. observed that approximately 30% reported the recommended level of physical activity.^24^ A Brazilian study showed that about 70% healthy adolescents have low levels of physical activity.^25^ However, these studies used different evaluation methods. In the present study, IPAQ short version was used, which is considered to be reproducible in adolescents older than 14 years old, but with limitations in smaller children. However, when compared to objective measures, such as the accelerometer, the short version tends to overestimate the levels of physical activity. Additionally, the IPAQ tries to classify the time spent in moderate and strenuous activities, which are of subjective understanding and may be difficult to be interpreted by children. This could explain our findings of low capacity in 6MWT, but not so low levels of physical activity measured by IPAQ.^26^

There were no differences between patients with acyanotic CHD and cyanotic CHD, but both groups showed less distance compared to the predicted. Also, correlation was found between the number of surgical procedures and Z score 6MWT.

Patients who undergo more surgical procedures are more often restricted in their daily activities and consequently restricted to the exercise, in addition to presenting more serious injuries that require multiple surgical procedures.

The higher C-reactive protein levels in acyanotic and cyanotic CHD patients may indicate some degree of residual hypoxemia, which could result in the release of inflammatory mediators, even after surgical correction. Similar results have been shown by Tomita et al. in individuals with CHD and hypoxemia.^27^

Another important point to consider is the great variability among the different kinds of congenital heart lesions. In the present study, there were two patients who underwent Fontan operations, and one of them did not complete the test. It is expected that acyanotic and well-corrected acyanotic and biventricular lesions would have a better functional performance. These individuals have the capacity of increasing pulmonary perfusion during exercise, as opposed to Fontan patients, who increase pulmonary ventilation, but not perfusion in the same levels.^28^ Other literature data are from individuals who had CHD when young and were evaluated in adulthood, showing no differences between the predicted and observed distance walked in the 6MWT.^29^ Since those patients were investigated when much older than in the present study, a direct comparison between studies is not possible.

Considering blood pressure, systolic blood pressure rarely exceeds 200 mmHg in the maximum effort in children and adolescents, and diastolic blood pressure rarely increases significantly, more frequently falling or staying in basal values. In the present study, the systolic blood pressure did not change after the 6MWT, and the diastolic blood pressure increased only in acyanotic CHD patients, which can be explained by the fact that the systolic blood pressure at rest is higher in these patients than in cyanotic CHD individuals.^30^

No differences in the degree of dyspnea and fatigue of lower limbs were observed after 6MWT, as assessed by the Borg scale, an instrument that allows a subjective evaluation of the intensity of exercise. Hommerding et al.^31^ evaluated patients with cystic fibrosis aged between six and 18 years, observing that children with greater functional impairment had more dyspnea. However, many factors can interfere with the subjective response, such as the instructions received, or understanding of the method and its purpose. It is likely that children older than nine years old, with a higher cognitive ability, understand better the scale. To minimize this factor, the scale used in the present study was colored, identifying the lowest degree of dyspnea as blue and the highest as red, in an attempt to facilitate the understanding of children.

The main limitation of this study was that CHD is a disease with low prevalence and high heterogeneity regarding exercise restrictions, prognosis and result of surgical/invasive interventions, thus making it difficult to evaluate a group as a whole. Individual characteristics must be accounted for, and larger groups of specific lesions are needed to issue clinical recommendations. Additionally, the instrument used to evaluate the level of physical activity was the IPAQ short version, that measures only physical activity intensity and time. The IPAQ long version evaluate different activities in various areas, such as work, travelling, leisure or domestic activities, and each of these domains is more explored than in the short version.^32^ Another limiting factor was the use of the 6MWT for evaluate the functional capacity, since this test is not the gold standard in this type of study, although it has good performance when compared with ergospirometry.^33^ However, the present study was important to increase the knowledge on physical capacity in this context, since the main objective was to use a simple and low-cost test in clinical practice, in order to increase awareness about the need of discussing thoroughly preventive measures to increase the levels of physical activity in this group.

In conclusion, children and adolescents with CHD are rarely completely sedentary, but the frequency of very active patients is low, featuring a population with less healthy lifestyle habits than usual in this age group. The functional capacity, lower than predicted for gender, age and height, is in agreement with this information, and is similar among individuals with cyanotic and acyanotic CHD. These findings are preliminary and should be confirmed in larger samples. Results showing that patients with cyanotic CHD can maintain some degree of inflammation in the long run, even after surgical correction, need to be confirmed in further studies. These results are relevant for the planning of preventive measures in this group of patients, mainly with regard to recommendations for physical activity of patients and guidance for the families according to the specific indications in each case.
